# The C(-1019)G 5-HT1A promoter polymorphism and personality traits: no evidence for significant association in alcoholic patients

**DOI:** 10.1186/1744-9081-2-7

**Published:** 2006-02-25

**Authors:** G Koller, B Bondy, UW Preuss, P Zill, M Soyka

**Affiliations:** 1 Psychiatrische Klinik der LMU, Substitutionsambulanz, Pestalozzistraße 2, D-80469 München, Germany; 2 Psychiatrische Klinik der LMU, Neurochemie, Nußbaumstraße 7, D-80336 München, Germany; 3 Johanna-Odebrecht-Stiftung, Gützkower Landstraße 69, D-17489 Greifswald, Germany; 4 Psychiatrische Klinik der LMU, Nussbaumstrasse 7, D-80336 München, Germany

## Abstract

The 5HT1A receptor is one of at least 14 different receptors for serotonin which has a role in moderating several brain functions and may be involved in the aetiology of several psychiatric disorders. The C(-1019)G 5-HT1A promoter polymorphism was reported to be associated with major depression, depression-related personality traits and suicidal behavior in various samples. The G(-1019) allele carriers are prone to depressive personality traits and suicidal behavior, because serotonergic neurotransmission is reduced.

The aim of this study is to replicate previous findings in a sample of 185 Alcohol-dependent individuals. Personality traits were evaluated using the NEO FFI and TCI. History of suicidal behavior was assessed by a standardized semistructured interview (SSAGA). No significant differences across C(-1019)G 5-HT1A genotype groups were found for TCI temperament and character traits and for NEO FFI personality scales. No association was detected between this genetic variant and history of suicide attempts. These results neither support a role of C(-1019)G 5-HT1A promoter polymorphism in the disposition of personality traits like harm avoidance or neuroticism, nor confirm previous research reporting an involvement of the G allele in suicidal behavior in alcoholics. Significant associations, however, were detected between Babor's Type B with number of suicide attempts in history, high neuroticism and harm avoidance scores in alcoholics.

## Background

The 5-hydroxytryptamine 1A receptor (5-HT1A) is one of several different receptors for 5-hydroxytryptamine (serotonin). Serotonin has a role in moderating several brain functions and is involved in the aetiology of several psychiatric disorders. Evidence for a role of 5-HT1A receptor in the pathophysiology of anxiety and depression has come from several clinical studies as well as from animal models [1-3]. In animal models, 5-HT1A receptor-deficient animals have been reported to have an increased level of anxiety and stress response as well as decreased exploratory activity [4,5]. The 5-HT1A receptor is also suggested to be involved in the response to antidepressant and anxiolytic drugs [6,7].

The 5-HT1A receptor is an integral membrane protein and belongs to the family of G-protein coupled receptors that inhibit adenylate cyclase activity. Negative feedback inhibition of serotonergic raphe neurons is mediated by somatodendritic 5-HT1A autoreceptors [8,9]. Reduced serotonergic neurotransmission is implicated in the pathogenesis of depressive illness and suicidal behaviors [10]. The gene for the 5-HT1A receptor is located at chromosomal region 5q11.2-q13 [11]. Human 5-HT1A gene transcription is modulated by a common C(-1019)G single nucleotide polymorphism (SNP) in its upstream regulatory region. The C(-1019) allele is part of a 26 bp imperfect palindrome that binds transcription factors NUDR (nuclear deformed epidermal auto regulatory factor) whereas the G(-1019) allele abolishes repression by NUDR, but only partially impairs Hes5-mediated repression. It was suggested that the G(-1019) allele derepresses 5-HT1A auto receptor expressions to reduce serotonergic neurotransmission [12].

Previous studies reported an association of the C(-1019)G 5-HT1A-promoter polymorphism with major depression and suicide in a number of samples.

There is a close relationship between the diagnosis depression and certain personality traits. The personality characteristic neuroticism and the temperament trait harm avoidance seem to be most interesting in this field. Neuroticism is described to be related with anxiety and depression [13]. Regarding temperament traits, harm avoidance have also been reported to be associated with anxiety and depression [14]. As there is a close relationship between character and personality traits to depressive and anxiety symptoms, the role of C(-1019)G 5-HT1A polymorphism in modulating these personality traits was evaluated to investigate the biological underpinnings of these important characteristics.

A significant effect of the C(-1019)G 5-HT1A polymorphism on neuroticism was detected with carriers of the G allele having higher scores than individuals homozygous for the C nucleotide. Carriers of the G allele also exhibited higher harm avoidance scores than carriers of the C allele [15].

Since suicidal behaviour is one of the diagnostic criteria for depression, a relationship between suicidal behavior and 5-HT1A receptor genetic variants can be expected but conflicting results were reported [16].

Persistent alterations in neuroendocrine responsiveness of both 5-HT and noradrenergic systems in alcoholic patients are documented [17]. Thus, an investigation of the influence of C(-1019)G 5-HT1A-promoter polymorphism on anxiety and depression-related personality traits in alcohol-dependent individuals might be of interest.

This manuscript represents an association study in a new population-German alcoholics. Suicide in a pure alcoholic sample is thought to have similar etiology to suicide in a depression sample.

The aim of our study is to investigate the C(-1019)G 5-HT1A-promoter polymorphism and its possible association with anxiety and depression-related personality traits and suicidal behavior. We hypothesize a significant effect of the C(-1019)G 5-HT1A polymorphism on neuroticism with carriers of the G allele having higher scores than individuals homozygous for the C variant as well as carriers of the G allele showing higher harm avoidance scores. Furthermore we hypothesize carriers of the G allele showing more suicide attempts in history. Since the severity of alcohol dependence and psychopathology differ in subtypes of alcoholics, potential interactions with Babor's Type A/ B subtypes were also investigatated.

## Methods

### Study design

From the addiction treatment ward of the psychiatric hospital at the University of Munich, 185 treatment-seeking individuals were recruited. All patients were unrelated, of German descent, older than 18 years and met ICD-10 and DSM-IV criteria for alcohol dependence, assessed with the structured clinical interview DSM IV [18] and SSAGA (Semi-Structured Assessment on the genetics in alcoholism) [19,20]. The diagnostic assessment was performed without knowledge of genotype data.

All patients were assessed two weeks after admission and after completing alcohol withdrawal, being free of any psychopharmacologic treatment for an average of 1.5 +0.6 weeks. Alcohol-dependent patients were excluded if they had a history of an independent major psychiatric disorder (occurring before the onset of alcohol dependence or during prolonged periods of abstinence) or substance dependence other than alcohol, marijuana or nicotine.

Informed written consent was obtained from patients after complete and extensive description of the study. The study was approved by the ethical committee of the Ludwig-Maximilians University of Munich in agreement with the principles laid down in the Helsinki Declaration (1964).

All participants completed the German version of Neo FFI and TCI [21,22]. The NEO Five-Factor Inventory is a shortened version of the NEO-PI, designed to give quick, reliable and valid measures of the five domains of adult personality including Neuroticism, Openness, Extraversion, Agreeableness and Consciousness. The 60 items are rated on a five-point scale. The NEO-FFI has a grade six reading level, and requires 10–15 minutes to complete [23]. The Temperament and Character Inventory (TCI) is a self-rating questionnaire designed to quantify individual differences on each of the temperament (Harm avoidance, Novelty seeking, Reward dependence and character dimension (Resistance, Self-directedness, Cooperation, Self-transcendence) [24]. Diagnostic and sampling procedure and research instruments were also described in previous publications [25,26].

Withdrawal symptomatology as well as a history of suicide attempts were evaluated using the SSAGA (Semi-Structured Assessment on the genetics in alcoholism [19,20].

Alcohol-dependent patients were classified as Type A or Type B using Babor Typology [27], using the criteria of Schuckit [28]. Characteristics of alcohol dependence, including the five characteristics mentioned by Schuckit were assessed using the SSAGA. These characteristics included daily alcohol intake (drinks of alcohol per drinking day (g/d), relief drinking, medical conditions due to alcohol, and physical and social consequences. Taking SSAGA data, composite scales were calculated for the five characteristics and dichotomised using median split. The 5 items were normalized prior to creating a composite scale. Subjects were subtyped according to Type A or B if they met three or more of the dichotomised scores criteria (upper half: Type B, lower half Type A).

### Genotyping

Genomic DNA was isolated from whole blood according to standard procedures. All genotyping was performed with the fluorescence resonance energy transfer method (FRET) using the Light Cycler System (Roche Diagnostics, Mannheim, Germany). A detailed description of the theoretical background and methodology is given [29]. For the C-1019G polymorphism in the 5HT1A receptor gene the following conditions were applied. Forward primer: 5'-CCG TTT TGT TGT TGT TGT CG-3'; reverse primer: 5'-CCA GCA AAA CTG GGG TTG3'; donor hybridization probe: 5'-TTT AAA AAC GAA GAC ACA CTC-fluorescein-3'; acceptor hybridization probe:

5'-LCRed640-CTT CTT CCA TCA ATT AGC AAT AAT TGG GAG-3'. PCR was performed with 50 ng DNA in a total volume of 20 μl containing 2 μl reaction mix, 0.5 μM each primer, 0.2 μM each hybridization probe and 2 μM MgCl_2 _according to the manufacturer's instructions for 35 cycles of denaturation (95°C, 0 s, ramp rate 20°C/s), annealing (55%°C, 10 s, ramp rate 20°C/s) and extension (72°C, 10 s, ramp rate 20°C/s). After amplification a melting curve was generated by holding the reaction at 40°C for 20 s and then heating slowly to 95°C with a ramp rate of 0.2°C/s. The fluorescence signal was plotted against temperature to give melting curves for each sample. Peaks were obtained at 57°C for the C-allele and at 46°C for the G-allele. All laboratory procedures were carried out blind to diagnostic assessment.

### Statistical analyses

To analyze relationships between personality traits and suicidal behavior with 5-HT1A genotypes and subtypes of alcoholics in a single model, a single MANOVA (multivariate analysis of variance) was performed. Suicide attempts in history, neuroticism and harm avoidance were used as dependent variables, C(-1019)G 5-HT1A and Babor Typology were used as independent factors. A p-value of <0.05 (2-tailed) was considered as statistically significant.

## Results

A total of 185 alcohol-dependent individuals were enrolled into the study. Sample characteristics are presented in table 1.

**Table 1:** Sample characteristics

	**Male**	**Female**
**Number**	150 (81,1%)	35 (18.9%)
**Mean Age**	41.6 ± 9,69	41,5 ± 3,56
Mean age of onset	28,56 ± 8,55	31,8 ± 8,77
Mean alcohol intake (g per day)	360,75 ± 199,8	216,15 ± 108,33
Type A	97 (52,4%)	25 (13,5%)
Type B	53 (28,6%)	10 (5,5%)
At last one suicide attempt in history	31 (16,7%)	10 (5,4%)

Using a single MANOVA no significant association was detected between C(-1019)G 5-HT1A and suicide attempt in history (F = 0.230, p = 0.795, df = 2), between C(-1019)G 5-HT1A and neuroticism (F = 0.811, p = 0.446, df = 2) and between C(-1019)G 5-HT1A and harm avoidance (F = 0.268, p = 0.765, df = 2).

However, significant associations were found between Babor's Type A/B and suicide attempts in history (F = 9.299, p = 0.003, df = 1), neuroticism (F = 9.857, p = 0.002, df = 1) and harm avoidance (F = 5.383, p = 0.022, df = 1). Type B shows higher neuroticism scores, as well as higher harm avoidance scores and a more frequent amount of suicide attempts in history.

No significant interaction between C(-1019)G 5-HT1A and Babor Typology in suicide attempts (F = 1.220, p = 0.298, df = 2), between C(-1019)G 5-HT1A and Babor Typology and neuroticism (F = 0.763, p = 0.468, df = 2) and between C(-1019)G 5-HT1A and Babor Typology and harm avoidance (F = 0.803, p = 0.450, df = 2) could be detected (s.table 2).

**Table 2:** Total numbers, Means of Neuroticism and Harm avoidance and numbers of patients with suicide attempts in history grouped by GG, GC and CC and by Babor Typology(SD = Standard deviation)

	**GG Type A**	**GG Type B**	**GC Type A**	**GC Type B**	**CC Type A**	**CC Type B**
**Number of patients**	30 (16,2%)	15 (8,1%)	62 (33,5%)	32 (17,2%)	30 (16,2%)	16 (8,6%)
**Neuroticism score (Mean/SD)**	22,6 ± 8	28,2 ± 8	23,3 ± 8	27,3 ± 8	22,1 ± 6	25,1 ± 8
**Harm avoidance score (Mean/SD)**	16,1 ± 6	19,7 ± 6	15,9 ± 6	18,0 ± 6	16,2 ± 6	17,4 ± 5
**Suicide attempts in history (Number of patients)**	4 (2,1%)	7 (3,7%)	11 (5,9%)	10 (5,4%)	3 (1,6%)	6 (3,2%)

## Discussion

The present study re-examined the potential association of a functional 5-HT1A-receptor polymorphism, a history of suicide attempts, and personality traits of harm avoidance and neuroticism. Our results do not support an association between the C(-1019)G 5-HT1A-promoter polymorphism with these personality and temperament traits, and also failed to detect an association between this functional polymorphism and a history of suicide attempts in 185 alcohol-dependent individuals. Significant associations were found between Babor's Type B and increased number of suicide attempts in history as well as higher neuroticism and harm avoidance scores.

The only previous study reporting an association between personality traits and this polymorphism in individuals of Caucasian background described the C(-1019)G 5-HT1A promoter polymorphism to be associated with the personality traits harm avoidance and neuroticism [15]. In accord with our assessments, in this prior study personality traits were assessed by NEO-PIR and TPQ.

The NEO FFI has five dimensions; their mean and standard deviation reference values in German population were described by authors of the German version [21]. The scale Harm avoidance is in line with a low score in self-directedness reflecting low responsibility, low purposefulness, low resourcefulness, low self-acceptance and low congruent second nature [30].

Thus, the divergent results deserve comments. The conceptual frame of anxiety-and depression-related personality traits and its assessment in alcoholics shortly after alcohol detoxification may be a relevant factor influencing the results.

The association of the G allele with certain personality traits may be different in subjects affected by other psychiatric disorders. For an association between suicide attempts and C(-1019)G 5-HT1A-promoter polymorphism the power of our sample might be too low (41 individuals reported a history of suicide attempts). Suicidal behaviour in general may be too heterogeneous to be a suitable phenotype to demonstrate genetic influence of single polymorphisms. Another limitation of the study is that participants were drug free only for 1,5 ± 0,6 weeks before the study but the 5-HT1A autoreceptor take 2–3 weeks to desensitise [31]. Reinvestigation of the participants after a longer drug free interval could be helpful.

Furthermore, it is not unusual that results of association studies significantly depend on the assessment method, i.e. the type of questionnaires and tests used. In a recent meta-analysis contrasts between groups were shown to be significant for TCI/TPQ Harm avoidance but not for NEO Neuroticism [32]. This may indicate more heterogeneity for the phenotype

Another meta-analysis reported significant effects on 5-HTTLPR. For studies using the Neuroticism scale of Costa and McCrae [33]. Findings do indicate that the effect, if present, is small.

There are also effects from other parts of the serotonergic system on the 5-HT1A receptor. In a very recent study it was demonstrated for the first time that a functional polymorphism in the 5-HTT gene, but not the 5-HT1A-receptor gene itself, affects 5-HT1A receptor availability in man. The 5-HT1A-receptor genotype did not show any significant effects on [11C]WAY 100635 binding [34]. These results may explain our results as far as functional significance is not as predicted.

Patients with panic disorder and depression exhibit an attenuation of 5-HT1A receptor-mediated neuroendocrine response, reflecting dysfunction of pre and postsynaptic 5-HT1A receptors [35]. Subsequent studies suggested the same mechanism to be associated with agoraphobia in a group of patients with panic disorders [36].

Decreased ligand binding of 5-HT1A receptors has been shown in depressed patients [37].

Not much data are available about association of personality traits and C(-1019)G 5-HT1A-promoter polymorphism. Regarding an association between the C(-1019)G 5-HT1A-promoter polymorphism and suicidal behavior, a recent study detected a significant association between this polymorphism and completed suicide [12]. However, the Pro16Leu and Gly272Asp polymorphisms of the 5-HT1A receptor gene failed to show an association with completed suicide in another study [38].

Since not much data are available in this field additional data may provide additional support or fail to support the hypothesis. Use of haplotypes for a particular gene or a broader assessment of serotonergic genes is mentioned as a stronger methodology.

## Conclusion

In conclusion our data failed to show the previously described association between the C(-1019)G 5-HT1A-promoter polymorphism and certain personality traits, which are depression- and anxiety-related. Our data also do not support an association between C(-1019)G 5-HT1A-promoter polymorphism and a history of suicide attempts. Further studies should address some of the open questions that remain. Another possible issue is whether therapeutic response to serotonergic agents is influenced by this polymorphism. Significant association is shown between Babor's Type B and number of suicide attempts in history, higher neuroticism and harm avoidance scores.

## Competing interests

The author(s) declare that they have no competing interests.

## Authors' contributions

G Koller participated in the design of the study and performed the statistical analysis and is writer of the text.

B Bondy and P Zill carried out the molecular genetic studies

U Preuss and M Soyka conceived of the study, and participated in its design and coordination and helped to draft the manuscript. All authors read and approved the final manuscript.
